# Effectiveness of non-pharmacological interventions for cognitive impairment in Parkinson’s disease: systematic review protocol

**DOI:** 10.1192/bjo.2025.10896

**Published:** 2025-11-07

**Authors:** Madeleine A. Homes-Vickers, David A. Hobbs, Anna V. Leonard, Lyndsey E. Collins-Praino

**Affiliations:** School of Biomedicine, Faculty of Health and Medical Sciences, The University of Adelaide, Adelaide, South Australia, Australia; College of Science and Engineering, Flinders University, Adelaide, South Australia, Australia; Allied Health & Human Performance, Innovation, IMPlementation and Clinical Translation (IIMPACT) in Health, University of South Australia, Adelaide, South Australia, Australia

**Keywords:** Cognitive impairment, non-pharmacological, Parkinson’s disease, therapy, treatment

## Abstract

**Background:**

Cognitive impairment is a significant, yet often overlooked, non-motor symptom of Parkinson’s disease, and a strong predictor of quality of life for those affected. Despite the availability of both pharmacological and non-pharmacological treatment options for Parkinson’s disease, their efficacy for the cognitive symptoms of the disease specifically is unclear, as no ‘gold standard’ treatment strategy for cognitive impairment in the disease has yet emerged. Further, a comparative understanding of the efficacy of each of these treatment options is severely lacking.

**Aims:**

This systematic review aims to critically evaluate the efficacy of non-pharmacological interventions for the treatment of cognitive impairment in Parkinson’s disease.

**Method:**

A comprehensive systematic search will be conducted to identify studies involving participants clinically diagnosed with Parkinson’s disease that assess non-pharmacological interventions targeting cognitive impairment. If feasible, results will be synthesised using meta-analysis; otherwise, narrative synthesis will be used.

**Results:**

This is a protocol for a systematic review that is yet to be conducted.

**Conclusions:**

The findings from this review will provide critical insight into the efficacy of non-pharmacological treatment options for cognitive impairment in Parkinson’s disease, which may help to influence clinical recommendations for the treatment of cognitive impairment in Parkinson’s disease and highlight existing gaps in the literature.

Parkinson’s disease is the second most common neurodegenerative condition, currently affecting more than 10 million individuals worldwide.^1^ Parkinson’s disease is also the fastest growing neurological condition, with an estimated 76% increase in prevalence expected by 2050.^2^ Although Parkinson’s disease is predominantly classified as a movement disorder, approximately 20% of individuals exhibit symptoms of cognitive impairment at the time of diagnosis, and this number increases to 40–50% at 5 years post-diagnosis.^3,4^

## Cognitive impairment

Many individuals with Parkinson’s disease experience single-domain cognitive decline, exhibiting impairments in attention, visuospatial function and executive function. However, cognitive impairment in Parkinson’s disease is heterogenous, with many individuals displaying cognitive decline across multiple or all domains.^5^ As such, cognitive impairment in Parkinson’s disease exists along a spectrum, ranging from normal cognition to mild cognitive impairment (MCI) and, ultimately, Parkinson’s disease dementia (PDD).^6^ As neurodegeneration related to Parkinson’s disease progresses, the prevalence of MCI also rises, with studies demonstrating that approximately 25–64% of individuals with Parkinson’s disease eventually develop MCI.^5,7^ For diagnosis of MCI, standardised diagnostic criteria developed by the Movement Disorder Society (MDS) are utilised across five cognitive domains (attention and working memory, language, memory, executive, and visuospatial function). Level I of these criteria requires the presence of cognitive deficits in global function on a measure validated for use in Parkinson’s disease, or impairment on at least two tests when a limited battery of neuropsychological tests is performed (e.g. fewer than two tests within each of the five cognitive domains or assessment of fewer than five cognitive domains).^8^ Conversely, level II of the MDS criteria requires comprehensive neuropsychological testing, including two tests within each of the five cognitive domains, with impairment within at least two of these (either two tests within one cognitive domain or one test in two different cognitive domains).^8^ The criteria used to define impairment vary among clinicians and research studies, with some relying on normative data (e.g. performance 1–2 standard deviations below norms) and others using significant decline from estimated premorbid levels. Decline on serial cognitive testing can also be used and is considered to be the current gold standard, but the availability of such data may be limited by financial and practical considerations.

MCI is traditionally considered to be a transitional stage between normal cognition and dementia; however, emerging evidence has demonstrated that MCI does not always progress to dementia, and that cognition can often revert into the normal range.^9^ A recent meta-analysis demonstrated this, with 28% of participants with Parkinson’s disease who had previously been previously diagnosed with MCI returning to normal cognitive function within 3 years.^10^ As such, the potential reversibility of MCI offers a valuable opportunity for interventions aimed at preventing or delaying the onset of dementia, as well as highlighting the importance of early detection and intervention.

In some cases, MCI may progress to PDD, resulting in a substantial decline in cognition and/or behaviour that interferes with activities of daily living.^11^ In fact, evidence suggests that up to 90% of individuals with Parkinson’s disease will develop dementia within 15–20 years of diagnosis.^12^ Diagnostic criteria for PDD requires cognitive impairment to develop at least 1 year following the onset of Parkinson’s disease symptoms and to be severe enough to affect daily life. Impairment must be associated with overall reductions in global cognitive function on a measure such as the Mini-Mental State Examination or Montreal Cognitive Assessment (MoCA), although studies disagree about the specific cut-off scores that should be used to indicate PDD.^13^ Further, impairment should be evident in at least two of the following cognitive domains: attention, executive function, visuospatial ability and memory.^14^

Cognitive impairment in Parkinson’s disease is associated with substantially increased healthcare costs, with the overall annual cost of medical visits, specialist care and additional support services reaching $65 000 per individual with moderate to severe Parkinson’s disease in Australia.^15^ In addition, cognitive decline has been linked to further reductions in quality of life and increased depression and disability,^16^ all of which significantly affect emotional well-being, social interactions and sense of independence.^17^ Moreover, caring for an individual experiencing cognitive impairment imposes a substantial burden on caregivers and can contribute to depression, social isolation and reduced personal quality of life.^18^

## Treatment options

Despite the significant burden of cognitive impairment in Parkinson’s disease, there remains a lack of effective treatment options, resulting in a crucial need for further research in this area. Current management strategies for cognitive impairment in individuals with Parkinson’s disease exclusively target symptomatic manifestations and do not offer substantial relief.^19^ Although pharmacological interventions such as cholinesterase inhibitors and memantine are commonly prescribed,^20^ cholinesterase inhibitors are associated with severe gastrointestinal side-effects,^21^ and memantine has demonstrated limited efficacy.^22^ Alternatively, non-pharmacological interventions such as physical therapy, cognitive training and lifestyle alterations have shown promise in addressing cognitive impairment related to Parkinson’s disease but have yielded inconsistent results.^23^

Furthermore, despite the growing burden of cognitive impairment in Parkinson’s disease, there has been limited research to directly compare and assess the efficacy of non-pharmacological treatments. This lack of comparative studies is concerning, as it is essential to determine the most effective treatments for managing cognitive impairment in Parkinson’s disease, to help inform clinical decision-making and formulate evidence-based guidelines.

## Study objectives

This systematic review aims to address this gap by providing the first comprehensive evaluation of current and emerging non-pharmacological interventions for cognitive impairment in Parkinson’s disease. To date, systematic reviews on the treatment of cognitive impairment in Parkinson’s disease have primarily focused on the impact of individual non-pharmacological interventions (e.g. exercise, neurostimulation or cognitive training), and there has been no direct comparison of such interventions. Through this review, we will evaluate the effectiveness of each treatment option, assess the quality of existing evidence and identify additional gaps in the literature. By addressing these questions, we aim to enhance understanding of the management of cognitive impairment in Parkinson’s disease and clarify the strengths and limitations of current therapeutic options, to provide treatment recommendations.

Our research questions are as follows.

- What are the current and emerging non-pharmacological interventions for cognitive impairment in individuals with Parkinson’s disease?
- How effective are these non-pharmacological interventions in addressing cognitive impairment in individuals with Parkinson’s disease?

## Method

This protocol was written in accordance with the Preferred Reporting Items for Systematic Review and Meta-Analysis (PRISMA) Protocols (PRISMA-P) statement and has been registered in the International Prospective Register of Systematic Reviews (PROSPERO; registration number CRD42024552138). The completed PRISMA-P Checklist is provided as Supplementary Material 1 available at https://doi.org/10.1192/bjo.2025.10896.

### Inclusion and exclusion criteria

#### Participants

Studies will be included if they involve adult participants formally diagnosed with Parkinson’s disease using established criteria, such as the UK Parkinson’s Disease Society Brain Bank Diagnostic Criteria and MDS Clinical Diagnostic Criteria.^24,25^ There will be no restrictions on the basis of participants’ sex, gender, or ethnicity, nor on disease severity, duration or age at time of disease onset. Studies including participants with comorbid mood disorders, such as depression and anxiety, will also be included, although this will be considered in the data synthesis. Participants with a neurological condition in addition to Parkinson’s disease, such as traumatic brain injury, stroke, tumours or aneurysm, will be excluded.

#### Cognitive assessment

To be eligible for inclusion in this systematic review, studies must assess cognitive function in individuals with Parkinson’s disease using at least one objective measure of cognition, administered by a trained professional, both pre- and post-intervention. Studies may include either global or multi-domain estimates of overall cognitive function or domain-specific assessments (Table 1). We will also include studies that categorise individuals on the basis of their cognitive status (e.g. normal cognition, MCI, PDD) using clinical cut-off scores and/or validated diagnostic criteria.^8^ Studies that assess cognition using only patient self-report data will be excluded.

**Table 1:** Examples of outcome measures eligible for inclusion in the systematic review

Outcome measure	Assessment tool
Global cognition	Montreal Cognitive Assessment, Mini-Mental State Examination, Dementia Rating Scale, Frontal Assessment Battery, Addenbrooke’s Cognitive Examination
Learning	Reinforcement learning task
Working memory	Digit span (forwards and backwards)
Processing speed	Inspection time
Memory	Wechsler Memory Scale, Rey Auditory Verbal Learning Test
Executive function	Trail Making Test, Stroop Test, Wisconsin Card Sorting Test
Visuospatial function	Rey–Osterrieth Complex Figure Test, Judgement of Line Orientation
Reasoning	Raven’s Progressive Matrices
Response inhibition	Stop-signal task, Go/NoGo task
Decision-making	Iowa Gambling Task

#### Treatment strategy

All studies must include assessment of a non-pharmacological treatment targeting cognitive impairment in individuals diagnosed with Parkinson’s disease. Interventions will be classified according to their broad category and further subcategorised if sufficient data are available. Examples of how interventions will be broken into subcategories are presented in Table 2.

**Table 2:** Examples of interventions eligible for inclusion and possible subcategories

Intervention	Subcategories
Exercise	Aerobic (cardio) versus muscle strengthening (strength/resistance) versus flexibility/balance; high-intensity interval training
Mindfulness/wellness	Yoga versus meditation versus mindful breathing
Cognitive training	Computerised versus manual; process-based versus strategy-based; games-based versus non-games-based
Neuromodulation	Transcranial direct current stimulation versus transcranial magnetic stimulation versus transcranial alternating current stimulation

#### Study design

All types of study designs (e.g. cross-sectional, cohort, case–control, longitudinal) will be eligible for inclusion, provided studies are peer-reviewed and published in English. Excluded publication types will include *in vitro* or preclinical *in vivo* studies, reviews (including systematic reviews), meta-analyses, grey literature, case reports, conference abstracts or proceedings, protocols, books, opinions or editorials, replies or commentaries, and theses or dissertations.

### Search strategy

A systematic search will be conducted across multiple electronic databases from inception, including MEDLINE (via PubMed), Embase (via Ovid), Scopus, PsycINFO, Cumulative Index to Nursing and Allied Health Literature (EBSCOhost) and Web of Science. The search strategy has been customised for each database to include relevant subject headings and MeSH terms. The search strategy will include keywords such as ‘Parkinson’s disease’, ‘non-pharmacological interventions’, ‘cognitive impairment’ and related synonyms. We also included terms and applied filters to exclude preclinical studies and non-English studies. The search strategies are provided as Supplementary Material 2.

We developed this search strategy in consultation with a medical research librarian who has expertise in medicine and psychology. The search will not be limited by publication year, and any amendments made to the search strategy will be documented on PROSPERO. Before initiating data synthesis, we will re-run all searches and perform backward and forward snowballing to include any eligible studies published between this date and the initial search date.^26^

### Study records

#### Data management

Following the database searches, identified citations will be exported to EndNote (version 20.1 for Windows; Clarivate Analytics, Pennsylvania, USA; https://endnote.com) and imported into Covidence Systematic Review Software (2014 for Windows; Veritas Health Innovation, Melbourne, Australia; www.covidence.org), where duplicates will be removed. Following this, title and abstract screening, full-text screening, data extraction and quality assessment will be completed in Covidence.

#### Study selection

Title and abstract screening will be conducted independently by the primary reviewer (M.A.H.-V.), and studies selected for a full-text review will be assessed against the inclusion and exclusion criteria by three independent reviewers (M.A.H.-V., Kristo Daminato and Lily Baxter). The primary reviewer (M.A.H.-V.) will screen all full-text articles, and the secondary reviewers (Kristo Daminato and Lily Baxter) will screen a randomly selected subset of articles (up to 20%). Reasons for exclusion during full-text screening will be documented, and reviewers will not be blinded to the studies. Any discrepancies or conflicts during the screening phase will be resolved through discussions among the reviewers, or, where necessary, by consulting an additional reviewer. At the full-text screening stage, interrater reliability will be quantified using Cohen’s kappa,^27^ with values reported in the final review. The results of the screening process will be visually displayed using a PRISMA flow diagram.

#### Data extraction

We have developed a review-specific extraction form, including 68 items, with relevant information according to the PRISMA-P guidelines. This includes study design, sample size, participant demographics (including baseline medication), intervention details, outcome measures and key findings. Our developed extraction form is provided as Supplementary Material 3. Data will be extracted from the specified studies by two independent reviewers, with a third reviewer consulted to resolve any discrepancies. All data will be extracted by the primary reviewer (M.A.H.-V.), and the secondary reviewer (Angus McNamara) will extract a maximum of 20% of all articles to ensure interrater reliability. If raw agreement is less than satisfactory (<85%), discussion between the reviewers will occur, and the second reviewer will independently extract an additional 10% of included studies. This process will be repeated until a satisfactory level of agreement (>85%) is reached.

Should a study include data on more than one intervention or cognitive domain, all relevant information will be extracted. Missing data will be addressed by contacting corresponding authors directly via email; the authors will be given 1 month to respond. A follow-up email will be sent if no response is received within the month. If authors are unresponsive or cannot provide the required information, the study will be excluded.

### Outcomes and prioritisation

This study aims to explore the effectiveness of non-pharmacological interventions for treating cognitive impairment in Parkinson’s disease. Included studies must employ at least one objective measure of cognition, administered by a trained professional both pre- and post-intervention to measure changes. Assessments may target any aspect of cognitive function (e.g. memory, executive function, learning, attention, processing speed, inhibition); however, assessments targeting executive function and attention will be prioritised owing to the decline in these domains that is frequently observed in individuals with Parkinson’s disease.^28,29^ Assessments in specific cognitive domains are preferred over global measures of cognition, which are typically used as screening tools. Notably, general measures of cognition are known to suffer from ceiling and floor effects.^30^ By prioritising domain-specific measures, we will also be able to examine whether a given treatment strategy has benefits with respect to specific aspects of cognitive function. In cases in which both general and domain-specific measures of cognition are available for a given study, only domain-specific measures will be included in meta-analyses.

Another consideration is the prioritisation of objective assessments (e.g. Go/NoGo Task) over clinician-rated assessments (e.g. MoCA), given the impact of rater bias and experience.^31^ For studies that include both objective and clinician-rated assessments, we will only include objective assessments. Further, as continuous measures are more sensitive to changes in cognition,^32^ continuous assessments (e.g. MoCA score) will be prioritised over categorical assessments of cognition (e.g. MCI, PDD). Again, for studies that include both continuous and categorical assessments of cognition, only continuous measures will be included.

### Risk of bias (quality) assessment

The quality of the included studies will be independently assessed by two reviewers (M.A.H.-V. and Angus McNamara) using an unmodified version of the Cochrane Risk Of Bias In Non-Randomised Studies – of Interventions, Version 2 (ROBINS-I V2) tool.^33^ This tool evaluates confounding, classification of interventions, selection of participants into the study, deviation from the intended intervention, missing data, measurement of the outcome and selection of the reported result. Randomised controlled trials that meet inclusion criteria will be evaluated using the Cochrane Risk of Bias 2 (RoB2) tool.^34^ This tool evaluates the randomisation process, deviation from the intended intervention, missing data, measurement of the outcome and selection of the reported result. Each potential source of bias will be categorised as low risk, moderate risk, serious risk or critical risk of bias. After the bias domains have been completed by reviewers, an overall risk-of-bias judgment will be calculated using Cochrane’s algorithm. The primary reviewer will assess the quality of all studies, and the secondary reviewer will assess 20% of randomly selected articles. The two reviewers will discuss each domain to reach agreement, and any discrepancies between the reviewers will be resolved through consultation with a third reviewer. To assess interrater agreement, the intraclass correlation (ICC) and 95% confidence interval will be calculated for each source of bias and overall bias.^35^ In terms of reliability, ICC values less than 0.5 indicate poor reliability, values between 0.5 and 0.75 indicate moderate reliability, values between 0.75 and 0.9 indicate good reliability, and values greater than 0.9 indicate excellent reliability.^36^ Therefore, if ICC values are below 0.75,^37^ a third reviewer will be included to reach a satisfactory level of agreement.

### Data synthesis

#### Study grouping and reporting

Data from included studies will be categorised by non-pharmacological intervention type and further grouped by cognitive domain. A minimum of two eligible studies per group will be required for analysis. If an appropriate number of studies show sufficient homogeneity in terms of study design, population and cognitive assessment, a meta-analysis will be conducted. The level of statistical heterogeneity will be determined using the *I* ^2^ test.^38^ All statistical analyses will be performed using R (version 3.6.0+ for Windows; R Foundation for Statistical Computing, Vienna, Austria; https://www.r-project.org). Results will be presented using forest plots to visualise effect sizes and confidence intervals. To assess publication biases, we will use both funnel plots and Egger’s test.^39^

#### Confidence in cumulative evidence

The overall confidence in evidence will be assessed using the Grading of Recommendations Assessment, Development and Evaluation (GRADE) summary of findings (GRADEpro Guideline Development Tool).^40^ The primary reviewer (M.A.H.-V.) will rate the overall GRADE for all studies as high, moderate, low or very low, depending on risk of bias (ROBINS-I V2/RoB2 tool), consistency (*I* ^2^ values), directness, precision and publication bias (Egger’s test).

#### Analysis plan

Studies will be analysed using standardised mean differences, calculated as Hedge’s *g*, with corresponding 95% confidence intervals for continuous outcomes. Hedge’s *g* will be calculated using R to ensure consistency. For categorical outcomes, odds and risk ratios will be used where appropriate.

Where feasible, we will run subgroup analyses to further probe our results. Proposed subgroups include:

- domain-specific versus global measures of cognition; and
- cognitive categorisation (Parkinson’s disease overall versus Parkinson’s disease MCI and/or PDD).

Where meta-analysis is not feasible, results will be presented in narrative form, with detailed synthesis of the results in both text and table form. For example, although it may not be feasible to adjust for baseline dopaminergic medication status in a meta-analysis, this information will be included as a narrative synthesis across studies, to provide context for interpretation of the results.

## Discussion

To our knowledge, this protocol outlines the first systematic review to evaluate the effectiveness of non-pharmacological interventions for cognitive impairment in patients with Parkinson’s disease. Despite the significant burden of cognitive impairment in Parkinson’s disease, current treatments remain focused on managing symptoms, and a gold-standard management protocol is still lacking. Whereas pharmacological treatment options for cognitive impairment in Parkinson’s disease are predominantly ineffective and can induce severe side-effects, non-pharmacological interventions display significant promise, highlighting the importance of further investigation. By systematically assessing the efficacy and quality of each currently available non-pharmaceutical treatment, this review seeks to deepen understanding of how various strategies may target cognitive impairment and whether this varies as a function of cognitive domain or an individual’s cognitive status. Strengths of this protocol include the focus on individual cognitive domains (rather than global cognition), as well as the consideration of non-pharmacological intervention types as specific subcategories. This allows a more nuanced analysis of potential benefits and consideration of any outstanding gaps and/or limitations in the literature. Along with this, the use of six separate electronic databases in the literature search provides a broad and comprehensive overview of current and emerging non-pharmaceutical interventions. Importantly, this enables identification of literature that may be missed using a more limited search strategy. We anticipate that our findings will contribute to both future research and clinical decision-making, ultimately contributing to more targeted, effective interventions to address cognitive impairment in Parkinson’s disease. Moreover, findings from this review may assist in formulating guidelines for the use of non-pharmacological treatment strategies for cognitive impairment in Parkinson’s disease. These insights may also have broader implications for age-related cognitive decline and neurodegenerative diseases such as Alzheimer’s disease, potentially driving improved outcomes across a range of conditions.

## Supporting information

Homes-Vickers et al. supplementary material 1 Homes-Vickers et al. supplementary material

Homes-Vickers et al. supplementary material 2 Homes-Vickers et al. supplementary material

Homes-Vickers et al. supplementary material 3 Homes-Vickers et al. supplementary material

## Data Availability

Data availability is not applicable to this article as no new data were created or analysed in this study.

## Supplementary material

The supplementary material is available online at https://doi.org/10.1192/bjo.2025.10896
