# Supplementary material for: Effectiveness of non-pharmacological interventions for cognitive impairment in Parkinson’s disease: systematic review protocol
Source: BJPsych Open. 2025 Nov 7;11(6):e268. doi: 10.1192/bjo.2025.10896 (PMC12641400; doi:10.1192/bjo.2025.10896)
Supplement: Homes-Vickers et al. supplementary material 3 — Homes-Vickers et al. supplementary material [file S205647242510896Xsup003.docx]

**Table 1: MEDLINE (PubMed)**

| **Search** | **Query** | **Records** |
| --- | --- | --- |
| **#1** | ("Parkinson Disease"[majr] OR "PD"[tiab] OR "parkinson*"[ti] OR "PD-D"[tiab] OR "PD-MCI"[tiab] OR "PD mild cognitive impairment"[tiab] OR “PD MCI”[tiab] OR "parkinson's disease mild cognitive impairment"[tiab] OR "parkinson's disease dementia"[tiab] OR "parkinson disease dementia"[tiab] OR "PD dementia"[tiab] OR "PDD"[tiab]) | **258,670** |
| **#2** | ("Cognitive treatment*"[ti] OR "cognitive therapy"[ti] OR "cognitive intervention*"[ti] OR “therapeutics”[Majr] OR "medicat*"[ti] OR "drug therap*"[Majr] OR "memantine"[Mesh] OR "memantine"[tiab] OR "rivastigmine"[Mesh] OR "rivastigmine"[tiab] OR "donepezil"[Mesh] OR "donepezil"[tiab] OR "galantamine"[Mesh] OR "galantamine"[tiab] OR "rasagiline"[tiab] OR "Atomoxetine Hydrochloride"[Mesh] OR "atomoxetine"[tiab] OR "cholinesterase inhibit*"[tiab] OR "Cholinesterase Inhibitors"[Mesh]) | **2,987,740** |
| **#3** | ("Non-pharmaceutical*"[ti] OR "cognitive train*"[tiab] OR "Cognitive Training"[Mesh] OR "Serious-Gam*"[tiab] OR "serious gam*"[tiab] OR "Virtual Reality"[tiab] OR "Virtual Reality"[Mesh] OR "rehabilitation"[Majr] OR "behavioural therap*"[ti] OR "behavioral therap*"[ti] OR "behavioural interven*"[ti] OR "behavioral interven*"[ti] OR "psychotherapy"[Majr] OR "occupational therapy"[Majr] OR "neurostimulation"[ti] OR "transcranial magnetic stimulat*"[ti] OR "transcranial direct current*"[ti] OR "physical therap*"[ti] OR "diet therap*"[Majr] OR "nutrition therap*"[ti] OR "nutritional interven*"[ti]) | **454,188** |
| **#4** | ("Cognitive Dysfunction"[Mesh] OR "cognit*"[tiab] OR "Cognition"[Mesh] OR "neuropsychological tests"[Mesh] OR "neuropsycholo*"[tiab] OR "learning"[tiab] OR "processing speed"[tiab] OR "social function*"[tiab] OR "language"[tiab] OR "attenti*"[tiab] OR "executive function*"[tiab] OR "memory"[tiab] OR "MOCA" OR "Montreal cognitive assessment" OR "MMSE" OR "mini-mental*" OR "mini mental*" OR "Mild Cognitive Impairment"[tiab] OR "Memory"[Mesh] OR "Attention"[Mesh] OR "visuo-spatial*"[tiab] OR "visuospatial"[tiab] OR "inhibition"[tiab] OR "inhibition, psychological"[MeSH] OR "information processing*"[tiab] OR "Language"[MeSH] OR "Executive Function"[MeSH] OR "decision-making"[tiab] OR "thinking"[MeSH] OR "Learning"[MeSH] OR "Problem solving"[MeSH] OR "digit span*" OR "corsi span*" OR "Frontal Assessment Battery" OR "rey auditory verbal learning test*" OR "wechsler adult intelligence scale*" OR "trail making test*" OR "Wisconsin Card Sorting Test" OR "Semantic fluency" OR "Phonemic fluency" OR "Wechsler memory scale" OR "stroop test*" OR "babcock test*" OR "Dementia Rating Scale" OR "judgment of line*" OR "raven*" OR "Raven's*" OR "Inspection Time" OR "Response Inhibition" OR "Task Switch*" OR "Cognitive Control" OR "Executive Control" OR "CANTAB" OR "cambridge neuropsychological test automated battery" OR "neurocog*"[tiab]) | **3,776,778** |
| **#5** | **#1 AND (#2 OR #3) AND #4** | **6,333** |

**Table 2: Embase (Ovid)**

| **Search** | **Query** | **Records** |
| --- | --- | --- |
| **#1** | exp *Parkinson disease or (“PD” or “parkinson*” or “parkinson* disease”).ti or (“parkinson* disease dementia” or “PDD” or “PD*MCI” or “parkinson* disease mild cognitive impairment” or “PD*mild cognitive impairment” or “PD dementia”).ti,ab | **198,741** |
| **#2** | ("memantine" or "rivastigmine" or "donepezil" or "galantamine" or "rasagiline" or "atomoxetine" or "cholinesterase inhibit*" or “pharmacological intervention*" or “therap*” or “treatment*” or “intervention”).ti or exp memantine or exp rivastigmine or exp donepezil or exp galantamine or exp rasagiline or exp atomoxetine or exp *cholinesterase inhibitor or exp *drug therapy | **981,525** |
| **#3** | ("cognitive train*" or “non*pharmaceutical” or “non*pharmacological” or "Serious-Gam*" or “serious gam*” or "Virtual Reality" or "non-pharmacological intervention*" or "neurostimulation" or "transcranial*”).ti or exp *cognitive rehabilitation or exp virtual reality or exp *rehabilitation or exp behaviour therapy or exp nerve stimulation or exp occupational therapy or exp physiotherapy or exp diet therapy | **971,443** |
| **#4** | (Cognitive dysfunction or cognitive decline or cognitive impairment).ti or (MoCA or MMSE or “mini*mental” or “visuospatial” or inhibition or “digit span*” or “corsi span*” or “frontal assessment battery” or “babcock test*” or “dementia rating scale” or “judgement” or “Raven*” or “inspection time” or “task switch” or “cognitive control” or “executive control” or “CANTAB” or “Cambridge neuropsychological test automated battery” or “semantic fluency” or “phonemic fluency” or “neurocog*”).ti,ab or exp *cognitive defect or exp cognition assessment or exp *cognition or exp neuropsychological assessment or exp memory or exp attention or exp learning or exp processing speed or exp social interaction or exp language ability or exp executive function or exp *mild cognitive impairment or exp Montreal cognitive assessment or exp cognitive impairment assessment or exp mini mental state examination or exp “inhibition (psychology)” or exp information processing or exp decision making or exp thinking or exp problem solving or exp Rey auditory verbal learning test or exp Wechsler intelligence scale or exp Wechsler adult intelligence scale or exp Wechsler memory scale or exp trail making test or exp Wisconsin Card Sorting Test or exp Stroop test or exp intelligence test | **6,453,423** |
| **#5** | #1 AND (#2 OR #3) AND #4 | **7,583** |

**Limited to Human studies, English and publication type = 6176**

**Table 3: PsychINFO**

| **Search** | **Query** | **Records** |
| --- | --- | --- |
| **#1** | exp *Parkinson’s disease or (“parkinson* disease dementia” or “parkinson*” or “parkinson* disease”).ti or (PD or PDD or PD*MCI or “parkinson* disease mild cognitive impairment” or PD*mild cognitive impairment or PD dementia).ti,ab | **40,140** |
| **#2** | ("memantine" or "rivastigmine" or "donepezil" or "galantamine" or "rasagiline" or "atomoxetine" or "cholinesterase inhibit*" or “pharmacological intervention*" or “therap*” or “treatment*” or “intervention”).m_titl or exp donepezil or exp galantamine or exp rasagiline or exp atomoxetine or exp *cholinesterase inhibitor or exp drug therapy | **452,864** |
| **#3** | ("cognitive train*" or “non*pharmaceutical” or “non*pharmacological” or "Serious-Gam*" or “serious gam*” or "Virtual Reality" or "non-pharmacological intervention*" or "neurostimulation" or "transcranial*”).m_titl or exp *cognitive rehabilitation or exp *virtual reality or exp rehabilitation or exp behavior therapy or exp nerve stimulation or exp occupational therapy or exp physiotherapy or exp diet therapy or exp treatment or exp intervention | **1,209,191** |
| **#4** | (Cognitive dysfunction or cognitive decline or cognitive impairment or MoCA or MMSE or “mini*mental” or “visuospatial” or inhibition or “digit span*” or “corsi span*” or “frontal assessment battery” or “babcock test*” or “dementia rating scale” or “judgement” or “Raven*” or “inspection time” or “task switch” or “cognitive control” or “executive control” or “CANTAB” or “cambridge neuropsychological test automated battery” or “semantic fluency” or “phonemic fluency” or “neurocog*”).m_titl or exp *cognitive impairment or exp cognitive assessment or exp *cognition or exp neuropsychological assessment or exp memory or exp attention or exp learning or exp processing speed or exp social interaction or exp language ability or exp executive function or exp *mild cognitive impairment or exp Montreal cognitive assessment or exp cognitive impairment assessment or exp mini mental state examination or exp “inhibition (psychology)” or exp information processing or exp decision making or exp thinking or exp problem solving or exp Rey auditory verbal learning test/ or exp Wechsler intelligence scale/ or exp Wechsler adult intelligence scale/ or exp Wechsler memory scale or exp trail making test or exp Wisconsin Card Sorting Test or exp Stroop test or exp intelligence test | **1,611,732** |
| **#5** | **#1 AND (#2 OR #3) AND #4** | **1,315** |

**Table 4: Web of Science**

| **Search** | **Query** | **Records** |
| --- | --- | --- |
| **#1** | TI=(“parkinson” or "parkinson? disease" or “PD”) or TS=(“PDD” or “PD MCI” or "PD-MCI" or “parkinson? disease mild cognitive impairment” or “PD?mild cognitive impairment” or “PD?dementia”) | **212,228** |
| **#2** | TS=("memantine" or "rivastigmine" or "donepezil" or "galantamine" or "rasagiline" or "atomoxetine") or TI=("cholinesterase inhibitor" or “pharmacological intervention" or “therapy” or “treatment” or “intervention” or "drug therapy") | **2,659,099** |
| **#3** | (TI=("cognitive train$" or “non$pharmaceutical” OR “non$pharmacological”)) OR (TS=("Serious$Gam" or "Serious Gam$" or "Serious-Gam$" or "Virtual Reality" or "VR" or "neurostimulation" or "transcranial” or "rehabilitation" or "behavioral therapy" "behavi$ral therapy" or "occupational therapy" or "physiotherapy" or "diet therapy")) | **534,884** |
| **#4** | (TI=("Cognitive dysfunction" or "cognitive decline" or "cognitive impairment" or "mild cognitive impairment")) OR (TS=("MoCA" or "MMSE" or “mini$mental” or “visuospatial” or "inhibition" or “digit span$” or “corsi span$” or “frontal assessment battery” or “babcock test$” or “dementia rating scale” or “judgement” or “Raven$” or “inspection time” or “task switch” or “cognitive control” or “executive control” or “CANTAB” or “Cambridge neuropsychological test automated battery” or “semantic fluency” or “phonemic fluency” or “neurocog$" or "cognition assessment" or "cognition" or "neuropsychological assessment" or "memory" or "attention" or "learning" or "processing speed" or "social interaction" or "language" or "executive function" or "Montreal cognitive assessment" or "cognitive impairment assessment" or "inhibition" or "information processing" or "decision making" or "thinking" or "problem solving" or "Rey auditory verbal learning test" or "Wechsler$" or "trail making test$" or "Wisconsin Card Sorting Test" or "Stroop test" or "intelligence test")) | **7,099,610** |
| **#5** | **#1 AND (#2 OR #3) AND #4** | **3,140** |

**Table 5: Scopus**

| **Search** | **Query** | **Records** |
| --- | --- | --- |
| **#1** | TITLE-ABS-KEY ( "PDD" OR "PD MCI" OR "PD-MCI" OR "PD" OR "parkinson* disease mild cognitive impairment" OR "PD*mild cognitive impairment" OR "PD*dementia" OR "parkinson*" OR "parkinson* disease" ) | **574,429** |
| **#2** | TITLE-ABS ( "memantine" OR "rivastigmine" OR "donepezil" OR "galantamine" OR "rasagiline" OR "atomoxetine" ) OR TITLE ( "cholinesterase inhibitor" OR "pharmacological intervention" OR "therapy" OR "treatment" OR "intervention" OR "drug therapy" ) | **3,125,824** |
| **#3** | TITLE-ABS ( "cognitive train*" OR "non*pharmaceutical" OR "non*pharmacological" ) OR TITLE-ABS-KEY ( "Serious*Gam" OR "Serious Gam*" OR "Serious-Gam*" OR "Virtual Reality" OR "VR" OR "neurostimulation" OR "transcranial" OR "rehabilitation" OR "behavioral therapy" "behavi*ral therapy" OR "occupational therapy" OR "physiotherapy" OR "diet therapy" ) | **102,338** |
| **#4** | TITLE ( "Cognitive dysfunction" OR "cognitive decline" OR "cognitive impairment" OR "mild cognitive impairment" ) OR TITLE-ABS ( "MoCA" OR "MMSE" OR "mini$mental" OR "visuospatial" OR "inhibition" OR "digit span$" OR "corsi span$" OR "frontal assessment battery" OR "babcock test$" OR "dementia rating scale" OR "judgement" OR "Raven$" OR "inspection time" OR "task switch" OR "cognitive control" OR "executive control" OR "CANTAB" OR "Cambridge neuropsychological test automated battery" OR "semantic fluency" OR "phonemic fluency" OR "neurocog$" OR "cognition assessment" OR "cognition" OR "neuropsychological assessment" OR "memory" OR "attention" OR "learning" OR "processing speed" OR "social interaction" OR "language" OR "executive function" OR "Montreal cognitive assessment" OR "cognitive impairment assessment" OR "inhibition" OR "information processing" OR "decision making" OR "thinking" OR "problem solving" OR "Rey auditory verbal learning test" OR "Wechsler$" OR "trail making test$" OR "Wisconsin Card Sorting Test" OR "Stroop test" OR "intelligence test" ) | **8,517,501** |
| **#5** | **#1 AND (#2 OR #3) AND #4** | **5,632** |

**Table 6: CINAHL**

| **Search** | **Query** | **Records** |
| --- | --- | --- |
| **#1** | (MM "Parkinson Disease") OR TI ( (“parkinson*” or "parkinson#disease" or “PD”) ) OR TI ( (“PDD” or “PD MCI” or "PD-MCI" or “parkinson#disease mild cognitive impairment” or “PD#mild cognitive impairment” or “PD#dementia”) ) OR AB ( (“PDD” or “PD MCI” or "PD-MCI" or “parkinson#disease mild cognitive impairment” or “PD#mild cognitive impairment” or “PD#dementia”)) | **30,839** |
| **#2** | ( (MH "Memantine") OR (MH "Rivastigmine") OR (MH "Donepezil") OR (MH "Galanthamine") OR (MH "Atomoxetine") OR (MM "Cholinesterase Inhibitors+") OR (MM "Drug Therapy") OR (MH "Prescriptions, Drug") ) OR TI ( ("memantine" OR "rivastigmine" OR "donepezil" OR "galantamine" OR "rasagiline" OR "atomoxetine") ) OR TI ( ("cholinesterase inhibitor" OR "pharmacological intervention" OR "therapy" OR "treatment" OR "intervention" OR "drug therapy") ) OR AB ( ("memantine" OR "rivastigmine" OR "donepezil" OR "galantamine" OR "rasagiline" OR "atomoxetine") ) | **600,952** |
| **#3** | ( (MM "Cognition+") OR (MH "Thinking") OR (MH "Mental Processes+") OR (MH "Cognitive Therapy+") OR (MH "Social Cognition") OR (MH "Rehabilitation, Cognitive") OR (MH "Virtual Reality+") OR (MH "Video Games+") OR (MM "Rehabilitation+") OR (MH "Behavior Therapy") OR (MH "Socioenvironmental Therapy") OR (MH "Psychotherapy+") ) OR TI ( ("cognitive train*" OR "non*pharmaceutical" OR "non*pharmacological") ) OR TI ( ( "Serious*Gam" OR "Serious Gam*" OR "Serious-Gam*" OR "Virtual Reality" OR "VR" OR "neurostimulation" OR "transcranial" OR "rehabilitation" OR "behavioral therapy" "behavi*ral therapy" OR "occupational therapy" OR "physiotherapy" OR "diet therapy" ) ) OR AB ( ( "Serious*Gam" OR "Serious Gam*" OR "Serious-Gam*" OR "Virtual Reality" OR "VR" OR "neurostimulation" OR "transcranial" OR "rehabilitation" OR "behavioral therapy" "behavi*ral therapy" OR "occupational therapy" OR "physiotherapy" OR "diet therapy" ) ) | **564,627** |
| **#4** | ( (MM "Mild Cognitive Impairment") OR (MM "Cognition Disorders") OR (MM "Cognition+") OR (MH "Neuropsychological Tests+") OR (MH "Language Tests") OR (MH "Memory and Learning Tests") OR (MH "Wisconsin Card Sorting Test") OR (MH "Word Lists") OR (MH "Thinking+") OR (MH "Learning+") OR (MH "Memory+") OR (MH "Problem Solving+") OR (MH "Perception+") OR (MH "Visual Perception+") OR (MH "Social Perception+") OR (MH "Decision Making+") OR (MH "Attention+") OR (MH "Executive Function") OR (MH "Language Processing") OR (MH "Wechsler Memory Scale-Revised") OR (MH "Wechsler Adult Intelligence Scale-Revised") OR (MH "Intelligence Tests") ) OR TI ( (Cognitive dysfunction or cognitive decline or cognitive impairment) ) OR TI ( (MoCA or MMSE or “mini*mental” or “visuospatial” or inhibition or “digit span*” or “corsi span*” or “frontal assessment battery” or “babcock test*” or “dementia rating scale” or “judgement” or “Raven*” or “inspection time” or “task switch” or “cognitive control” or “executive control” or “CANTAB” or “cambridge neuropsychological test automated battery” or “semantic fluency” or “phonemic fluency” or “neurocog*” or "cognition assessment" or "cognition" or "neuropsychological assessment" or "memory" or "attention" or "learning" or "processing speed" or "social interaction" or "language" or "executive function" or "Montreal cognitive assessment" or "cognitive impairment assessment" or "inhibition" or "information processing" or "decision making" or "thinking" or "problem solving" or "Rey auditory verbal learning test" or "Wechsler$" or "trail making test$" or "Wisconsin Card Sorting Test" or "Stroop test" or "intelligence test") ) OR AB ( (MoCA or MMSE or “mini*mental” or “visuospatial” or inhibition or “digit span*” or “corsi span*” or “frontal assessment battery” or “babcock test*” or “dementia rating scale” or “judgement” or “Raven*” or “inspection time” or “task switch” or “cognitive control” or “executive control” or “CANTAB” or “cambridge neuropsychological test automated battery” or “semantic fluency” or “phonemic fluency” or “neurocog*” or "cognition assessment" or "cognition" or "neuropsychological assessment" or "memory" or "attention" or "learning" or "processing speed" or "social interaction" or "language" or "executive function" or "Montreal cognitive assessment" or "cognitive impairment assessment" or "inhibition" or "information processing" or "decision making" or "thinking" or "problem solving" or "Rey auditory verbal learning test" or "Wechsler$" or "trail making test$" or "Wisconsin Card Sorting Test" or "Stroop test" or "intelligence test") ) | **916,606** |
| **#5** | **#1 AND (#2 OR #3) AND #4** | **1,414** |
